# Antibiotic Susceptibility, Genetic Diversity, and the Presence of Toxin Producing Genes in *Campylobacter* Isolates from Poultry

**DOI:** 10.3390/ijerph14111400

**Published:** 2017-11-17

**Authors:** Jeeyeon Lee, Jiyeon Jeong, Heeyoung Lee, Jimyeong Ha, Sejeong Kim, Yukyung Choi, Hyemin Oh, Kunho Seo, Yohan Yoon, Soomin Lee

**Affiliations:** 1 Department of Food and Nutrition, Sookmyung Women’s University, Seoul 04310, Korea; fmjylee02@naver.com (J.L.); jjy5654@naver.com (J.J.); hayan29@naver.com (J.H.); 3337119@hanmail.net (S.K.); lab_yukyung@naver.com (Y.C.); odry0731@naver.com (H.O.); 2 Risk Analysis Research Center, Sookmyung Women’s University, Seoul 04310, Korea; hylee06@sookmyung.ac.kr; 3 Department of Veterinary Medicine, Konkuk University, Seoul 05029, Korea; bracstu3@konkuk.ac.kr

**Keywords:** *Campylobacter*, poultry, antibiotic susceptibility, Rep-PCR, *cdt* toxin

## Abstract

This study examined antibiotic susceptibility, genetic diversity, and characteristics of virulence genes in *Campylobacter* isolates from poultry. Chicken (*n* = 152) and duck (*n* = 154) samples were collected from 18 wet markets in Korea. *Campylobacter* spp. isolated from the carcasses were identified by PCR. The isolated colonies were analyzed for antibiotic susceptibility to chloramphenicol, amikacin, erythromycin, tetracycline, ciprofloxacin, nalidixic acid, and enrofloxacin. The isolates were also used to analyze genetic diversity using the DiversiLab^TM^ system and were tested for the presence of cytolethal distending toxin (*cdt*) genes. *Campylobacter* spp. were isolated from 45 poultry samples out of 306 poultry samples (14.7%) and the average levels of *Campylobacter* contamination were 22.0 CFU/g and 366.1 CFU/g in chicken and duck samples, respectively. Moreover, more than 90% of the isolates showed resistance to nalidixic acid and ciprofloxacin. Genetic correlation analysis showed greater than 95% similarity between 84.4% of the isolates, and three *cdt* genes (*cdtA*, *cdtB*, and *cdtC*) were present in 71.1% of *Campylobacter* isolates. These results indicate that *Campylobacter* contamination should be decreased to prevent and treat *Campylobacter* foodborne illness.

## 1. Introduction

*Campylobacter* spp. are Gram-negative, microaerophilic bacteria, and the most common cause of bacterial foodborne illness in the world [1,2,3,4]. Among 17 *Campylobacter* species, *Campylobacter jejuni* and *Campylobacter coli* are the major causative agents of foodborne illness in human [5,6,7]. Animal species such as chicken, cattle and wild birds are reservoirs for *Campylobacter* [8,9]. *Campylobacter* infection causes watery diarrhea, fever, bloody stools, abdominal pain, and some complications such as Guillain-Barré syndrome (GBS) and Reiter’s syndrome in severe case [10]. Facciolà et al. [10] suggested that it is difficult to find the contamination sources because *Campylobacter* outbreaks were sporadic and caused by cross-contamination.

Recently, campylobacteriosis have increased dramatically in South Korea. Until 2002, there were no *Campylobacter* outbreaks, but 831 people were infected by *Campylobacter* in 2016 [11]. In Switzerland, campylobacteriosis have also been increased, and healthcare cost for the patients was $7.5 million per year, expected to increase steadily [12]. *Campylobacter* have several virulence factors such as flagellin, capsular polysaccharides, and cytotoxins [13]. Regarding cytotoxin production, *Campylobacter* can produce cytolethal distending toxin (CDT), which is encoded by *cdtA*, *cdtB,* and *cdtC* genes [14,15,16]. This toxin can induce the host cell distension, then lead to cell death [17]. In severe cases, antibiotic (erythromycin, ciprofloxacin, tetracycline, etc.) treatment is necessary to treat *Campylobacter* infection, but *Campylobacter* spp. have recently begun to show resistance to several antibiotics [18,19,20]. In a previous study, 159 *Campylobacter* isolates from poultry samples in China were examined for antibiotic resistance and 94% (149 isolates) of *Campylobacter* isolates were resistant to tetracycline, doxycycline, and erythromycin [18]. Thus, *Campylobacter* isolates need to be investigated for antibiotic susceptibility.

To analyze the genetic correlation among bacterial isolates, restriction-based, amplification-based, and sequencing-based methods have been used [21]. Restriction-based methods include plasmid analysis, restriction fragment length polymorphism (RFLP) analysis, and pulsed-field gel electrophoresis (PFGE). Amplification-based methods are amplified fragment length polymorphisms (AFLP), random amplified polymorphic DNA PCR (RAPD-PCR), and repetitive element PCR (Rep-PCR). Sequencing-based methods include multilocus sequence typing (MLST) and single-nucleotide polymorphism (SNP) analysis. Rep-PCR can assign molecular fingerprints according to the repetitive sequences in bacterial genomes [22,23]. Compared to other PCR typing methods, Rep-PCR has advantages: processing is rapid and it has the ability to analyze small amounts of DNA [21,24]. Abay et al. [25] also suggested that Rep-PCR was more powerful for typeability of *Campylobacter* than PFGE.

The objective of this study was to investigate the prevalence of *Campylobacter* in poultry carcasses in wet markets, determine antibiotic susceptibility patterns, the presence of *cdt* genes, and analyzed the genetic diversity between the *Campylobacter* isolates.

## 2. Materials and Methods

### 2.1. Sample Collection

**Figure 1:**
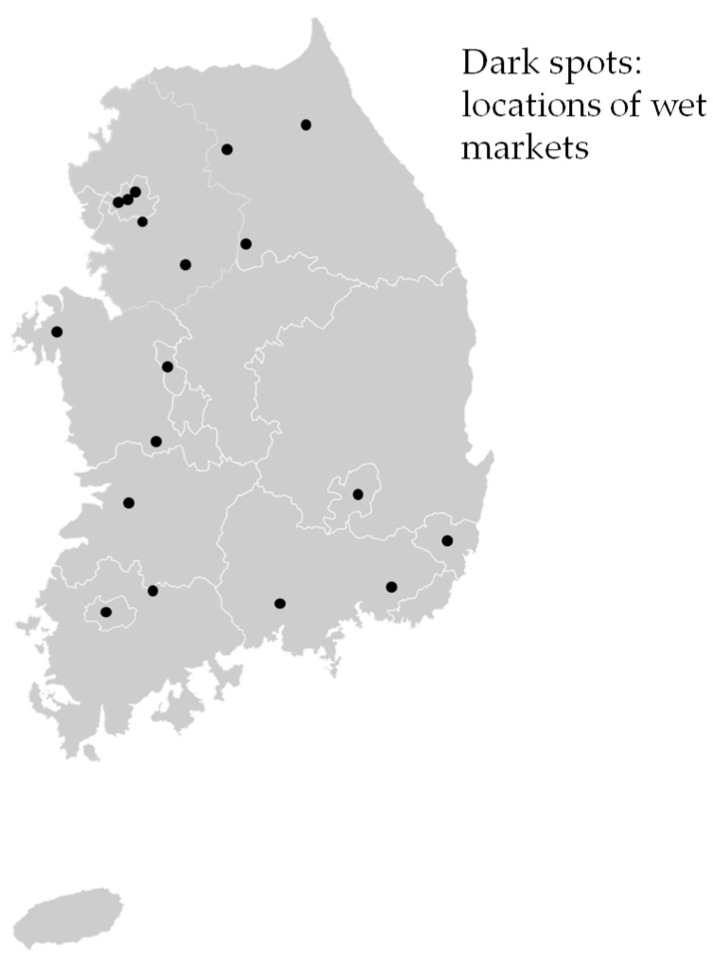
The locations of wet markets for poultry samples collected in Korea.

Chicken (*n* = 152) and duck (*n* = 154) carcasses were purchased from 18 wet markets throughout Korea during the summer (June–August, in 2014) and winter seasons (December in 2014 to February in 2015) (Figure 1). Three to ten samples for both chicken and duck carcasses were collected per market and per visit, and each market was visited twice for summer and winter. The samples were placed in a cooler on ice and transported to a laboratory. They were analyzed within 24 h.

### 2.2. Campylobacter Isolation, Enumeration, and Identification

Each poultry sample was placed into a sample bag containing 400 mL 0.1% buffered peptone water (BPW, Becton, Dickinson and Company, Sparks, MD, USA) and gently shaken for 60 s. For *Campylobacter* isolation, the rinsate (27 mL) was mixed with 27 mL 2 × blood-free Bolton broth (Oxoid Ltd., Basingstoke, Hampshire, UK) and the mixture was enriched at 42 °C for 48 h. Loopful portions (10 μL) of the enrichments were streaked on modified charcoal-cefoperazone-deoxycholate agar (mCCDA; Oxoid Ltd., Basingstoke, UK) and incubated at 42 °C for 48 h in a microaerobic environment (5% O_2_, 10% CO_2_, and 85% N_2_) created by CampyGen^TM^ gas packs (Oxoid Ltd., Basingstoke, UK). The two presumptive *Campylobacter* colonies (gray, mucoid, and flat) on a plate were selected and each colony of them was streaked on two Colombia agar plates (bioMérieux, Marcy-l’Étoile, France) for aerobic and microaerobic conditions at 42 °C for 48 h under both aerobic and microaerobic conditions. The colonies grown under microaerobic conditions were further analyzed to identify *Campylobacter* by PCR using the primers listed in Table 1. To extract *Campylobacter* DNA, the presumptive colonies at plate were suspended in 0.2 mL of sterilized distilled water, and heated at 99 °C for 10 min. The suspensions were centrifuged at 14,000 rpm for 3 min, and supernatants were then used for PCR amplification. The program was as follows: pre-denaturation at 95 °C for 15 min, 25 cycles of denaturation at 95 °C for 0.5 min, annealing at 58 °C for 1.5 min, and extension at 72 °C for 1 min. A final extension step at 72 °C for 7 min was performed [26]. The PCR products were visualized by electrophoresis and UV-transillumination. The isolates were used in further experiments for analysis of antibiotic resistance, genetic diversity and *cdt* genes. To enumerate *Campylobacter* cells, 1 mL of the rinsate was serially diluted using 0.1% BPW, and 0.1 mL of aliquots were plated on mCCDA (Oxoid Ltd., Basingstoke, UK). The plates were then incubated at 42 °C for 48 h under microaerobic conditions. Five presumptive colonies on each plate were then analyzed by PCR using the conditions described above. The contamination levels of *Campylobacter* were determined by multiplying the number of positive colonies per five presumptive colonies to the total number of colonies. Additionally, each carcass was weighted to calculate the colony forming units per g (CFU/g).

### 2.3. Antibiotic Susceptibility Testing

The isolated colonies were further analyzed for antibiotic susceptibility to chloramphenicol, amikacin, erythromycin, tetracycline, ciprofloxacin, nalidixic acid, and enrofloxacin (Sigma-Aldrich, St Louis, MO, USA), according to the guidelines of the Clinical & Laboratory Standards Institute [29]. To determine antibiotic resistance, the breakpoints suggested by CLSI [29], CDC [30], Hong et al. [31], and Kang et al. [32] were used as follows: chloramphenicol at 32 μg/mL, amikacin at 64 μg/mL, erythromycin at 32 μg/mL, tetracycline at 16 μg/mL, ciprofloxacin at 4 μg/mL, nalidixic acid at 64 μg/mL, and enrofloxacin at 4 μg/mL. The *Campylobacter* isolates on Colombia agar (bioMérieux, Marcy-l’Étoile, France) were suspended in Mueller-Hinton broth (MHB; Becton, Dickinson and Company, Sparks, MD, USA) to obtain a McFarland 0.5 standard, and further diluted 10-fold. Using needles, *Campylobacter* isolates were spotted on Mueller-Hinton agar (MHA; Becton, Dickinson and Company, Sparks, MD, USA) with 5% lysed horse blood plates (Oxoid Ltd., Basingstoke, UK), formulated at 0.5–128 μg/mL with seven antibiotics. The plates were incubated under microaerobic conditions at 37 °C for 48 h. MIC was determined by colony formation on the plates and the reference strain used was *Campylobacter jejuni* ATCC33560.

### 2.4. Analysis of Genetic Diversity

To analyze the genetic diversity, 45 *Campylobacter* isolates from poultry were streaked on Colombia agar (bioMérieux, Marcy-l’Étoile, France), followed by microaerobic incubation at 42 °C for 48 h. DNA was extracted from *Campylobacter* isolates using a commercial kit (UltraClean^TM^ Microbial DNA Isolation Kit, MoBio Laboratories, Solana Beach, CA, USA). The extracted DNA was amplified using DiversiLab *Campylobacter* Kit (bioMérieux, Marcy-l’Étoile, France). The amplified products were separated by electrophoresis on microfluidics chips (Agilent Technologies, Palo Alto, CA, USA) and analyzed with the Agilent 2100 Bioanalyzer (Agilent Technologies, Palo Alto, CA, USA). The peak and band data were analyzed by DiversiLab^TM^ software version 2.1.66 (bioMérieux, Marcy-l’Étoile, France) using Pearson’s correlation coefficient and unweighted pair group method with the arithmetic mean, followed by dendrogram generation. The cutoff value was 95% for determining genetic similarity [33,34].

### 2.5. Analysis of Cytolethal Distending Toxin Genes

To observe the presence of *cdt* genes (*cdtA*, *cdtB*, and *cdtC*) from isolates, the extracted DNA was amplified using the primers listed in Table 2 [14]. The PCR products were visualized by gel electrophoresis and UV-transillumination.

### 2.6. Statistical Analysis

The data for the prevalence and contamination levels of *Campylobacter* between chicken and duck were statistically analyzed by SAS version 9.3 (SAS Institute Inc., Cary, NC, USA), and Chi-square test and *t*-test were used for prevalence and contamination levels, respectively, to determine significance at α = 0.05.

## 3. Results and Discussion

### 3.1. Prevalence and Contamination Levels of Campylobacter

Of 306 poultry samples, *Campylobacter* spp. were identified from 45 samples (14.7%, 15 chicken samples and 30 duck samples) after enrichment (qualitative), but the number of positive samples was higher in quantitative results than in qualitative samples (Table 3). Since other bacteria may also be enriched with *Campylobacter*, resulting in disturbing the identification, the prevalence rate was lower in qualitative results than in quantitative results. The mean contamination levels of the isolated *Campylobacter* spp. in chicken and duck samples were 22.0 ± 36.3 CFU/g and 366.1 ± 733.6 CFU/g, respectively (Table 3).

These results suggest that a quantitative method may be appropriate to investigate *Campylobacter* prevalence rather than a qualitative method, and duck samples have a higher contamination frequency and have higher levels of contamination significantly (*p* = 0.0210) than those in chicken samples in the Korean markets. *Campylobacter* was isolated regardless of the season; however, the contamination levels of *Campylobacter* were higher in the winter than in the summer. Of the 45 *Campylobacter* spp. isolates, 29 isolates were *C. jejuni* and 16 isolates were *C. coli.* In France, 372 of 425 chicken samples (87.5%) were *Campylobacter* positive, and their mean contamination level was 2.4 log CFU/g [35]. Also, Garin et al. [36] showed that *Campylobacter* spp. were detected from 491 of 750 chicken carcasses (65.5%) in five countries (Senegal, Cameroon, Madagascar, New Caledonia and Vietnam), and the mean value of contamination level was 3.2 log CFU/g. Additionally, Zhu et al. [37] analyzed 1587 chicken carcasses collected from seven provinces in China, and 716 carcasses (45.1%) were contaminated to Campylobacter, and the contamination level was 2.1 log CFU/g (median value). These studies indicate that *Campylobacter* contamination levels were similar among countries, however, the prevalence of *Campylobacter* can be considered low in wet markets in Korea. *Campylobacter* are microaerophilic bacteria. Thus, the bacterial cell counts can be gradually decreased under aerobic condition during distribution. Hence, long exposure time to aerobic condition during distribution to wet markets may induce low prevalence of *Campylobacter* in poultry in Korea.

### 3.2. Antimicrobial Resistance Patterns

Because antimicrobial resistance patterns were not different between *C. jejuni* and *C. coli*, the data were combined in Table 4. The *Campylobacter* isolates were resistant to nalidixic acid (93.3%), ciprofloxacin (91.1%), and tetracycline (71.1%) (Table 4). The isolates showed especially strong resistance to antibiotics such as nalidixic acid ciprofloxacin, tetracycline. However, *Campylobacter* isolates were sensitive to chloramphenicol (others), enrofloxacin (fluoroquinolones), erythromycin (macrolides), and amikacin (aminoglycosides) (Table 4). In Italy, *Campylobacter* isolates also showed high resistance rates to ciprofloxacin, tetracycline, and nalidixic acid [38]. Similarly, in the USA, the rate of antimicrobial resistance to tetracycline was very high, at 99.1% in *Campylobacter* isolates from broiler carcasses, followed by resistance to nalidixic acid and ciprofloxacin [39].

Raeisi et al. [40] showed that *Campylobacter* isolates from poultry were resistant to ciprofloxacin, tetracycline and nalidixic acid. Also, 100% of *C. jejuni* isolates (*n* = 31) from chicken in China had resistance to ciprofloxacin and nalidixic acid [41]. In Poland, *Campylobacter* isolates were susceptible to erythromycin and resistant to tetracycline and ciprofloxacin [42]. Taken together, we can conclude that both poultry and human isolates of *Campylobacter* spp. are generally resistant to quinolone and fluoroquinolone antibiotics, such as nalidixic acid and ciprofloxacin. This may be caused by the use of these antibiotics in veterinary and human medicine. Therefore, this result suggests that antibiotics used for humans should not be used in poultry.

### 3.3. Genetic Diversity between Isolates

**Figure 2:**
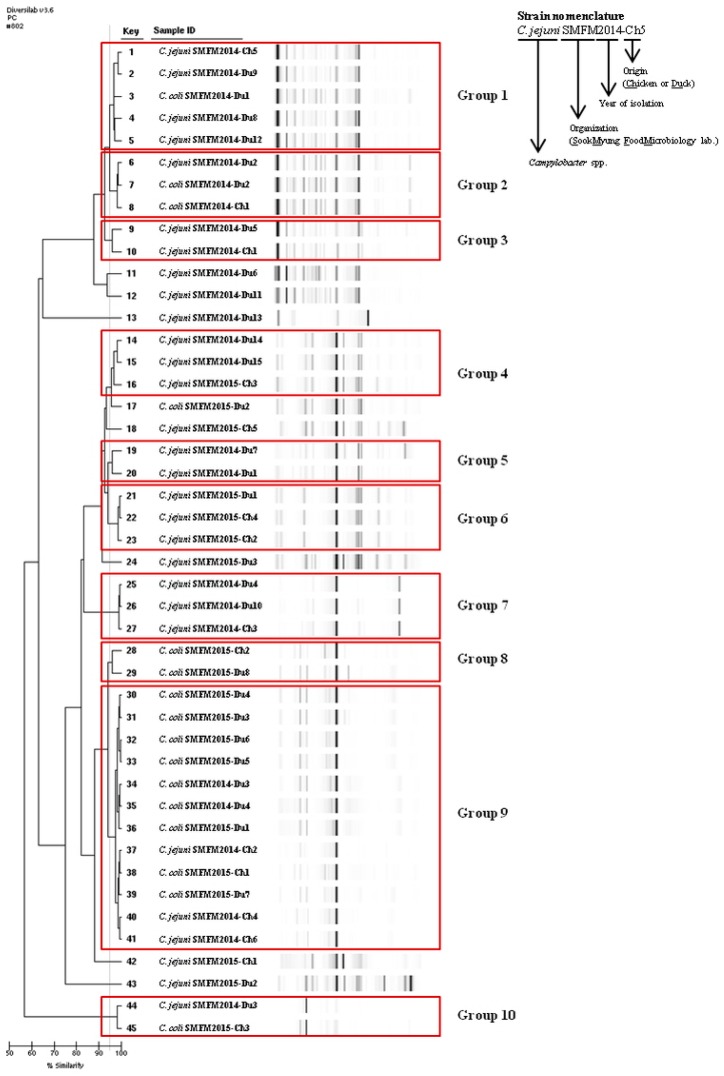
Dendrogram and gel-like image of the DiversiLab systems for *Campylobacter* isolates from poultry samples.

*Campylobacter* isolates were group according to the Rep-PCR dendrogram patterns (Figure 2). In genetic diversity, more than 95% similarity was shown in 38 isolates (84.4%) and these isolates were grouped into 10 groups (Figure 2). When comparing the 10 groups, obvious geographic correlations were not observed (Figure 2). For instance, key numbers 21–23 in group 6 were isolated from same location (Ulsan). Although 26–27 in group 7, and 39–41 in group 9 were isolated from same location (Cheongju), they were placed in different genetic group. However, Hiett et al. [43] subtyped for 50 *Campylobacter* isolates, and the most isolates from same location were genetically very similar. Like this result, very close genetic similarity can be expected for the isolates from same locations, but it was not observed in Korea as discussed above. This result indicates that chicken and duck in different wet markets in Korea may be distributed from only few slaughterhouses.

### 3.4. Distribution of cdtA, cdtB, and cdtC

*Campylobacter* can produce CDT, composed of A, B, and C subunits, which are encoded by *cdtA*, *cdtB*, and *cdtC* genes [44]. The 71.1% of the *Campylobacter* isolates had these three genes (Table 5). Nine of 15 chicken *Campylobacter* isolates and 23 of 30 duck *Campylobacter* isolates had the three *cdt* genes. Four isolates were found to be without any *cdt* genes and nine isolates had two *cdt* genes (*cdtA+*/*cdtB+*, *cdtA+*/*cdtC+*, or *cdtB+*/*cdtC+*). There was no relationship between the distribution of *cdt* genes and the regions the isolates had been obtained from. Oh et al. [45] showed that 37 *C. jejuni* isolates out of 38 chicken samples had all *cdt* genes. Findik et al. [5] found that 75.6% of *C. jejuni* isolates (127 isolates out of 168) from various sources, including human, poultry, cattle, sheep, and dog, had all *cdt* genes and five isolates were without *cdt* genes. In Brazil, all *cdt* genes were detected in 66.7% of *Campylobacter* isolates [46]. These results indicate that most *Campylobacter* isolates from our study have the potential to produce CDT.

## 4. Conclusions

In this study, the prevalence of the pathogen, antibiotic resistance, genetic diversity, and the presence of *cdt* genes in *Campylobacter* isolates were identified from poultry in Korean wet markets. Although the prevalence of *Campylobacter* in poultry was relatively low compared to that in other countries, antibiotic resistance patterns of the isolates were similar to those in other countries. In addition, geographic genetic diversity was not observed and a high proportion of *cdt* genes were present in *Campylobacter* isolates. Therefore, *Campylobacter* contamination should be decreased in order to prevent and treat the *Campylobacter* foodborne illness.

## Figures and Tables

**Table 1:** Primer sequences used to identify the *Campylobacter* genus and species.

Species	Target Gene	Primer	Sequence (5’→3’)	Size (bp)	Reference
Genus *Campylobacter*	16S rRNA	C412F	GGATGACACTTTTCGGAGC	816	[27]
		C1228R	CATTGTAGCACGTGTGTC		
*Campylobacter jejuni*	*cj0414*	C-1	CAAATAAAGTTAGAGGTAGAATGT	161	[28]
		C-3	CCATAAGCACTAGCTAGCTGAT		
*Campylobacter coli*	*ask*	CC18F	GGTATGATTTCTACAAAGCGAG	502	[27]
		CC519R	ATAAAAGACTATCGTCGCGTG		

**Table 2:** PCR primers and amplification conditions used to analysis of *cdt* genes for *Campylobacter* isolates.

Genus	Gene	Sequence (5’→3’)	Amplification ^(1)^ Condition	Size (bp)
*Campylobacter jejuni*	*cdtA*	F: AGGACTTGAACCTACTTTTC	94 °C, 30 s −55 °C, 30 s −72 °C, 30 s	631
		R: AGGTGGAGTAGTTAAAAACC		
	*cdtB*	F: ATCTTTTAACCTTGCTTTTGC	94 °C, 30 s −56 °C, 30 s −72 °C, 30 s	714
		R: GCAAGCATTAAAATCGCAGC		
	*cdtC*	F: TTTAGCCTTTGCAACTCCTA	94 °C, 30 s −55 °C, 30 s −72 °C, 30 s	524
		R: AAGGGGTAGCAGCTGTTAA		
*Campylobacter coli*	*cdtA*	F: ATTGCCAAGGCTAAAATCTC	94 °C, 30 s −55 °C, 30 s −72 °C, 30 s	329
		R: GATAAAGTCTCCAAAACTGC		
	*cdtB*	F: TTTAATGTATTATTTGCCGC	94 °C, 30 s −56 °C, 30 s −72 °C, 30 s	413
		R: TCATTGCCTATGCGTATG		
	*cdtC*	F: TAGGGATATGCACGCAAAAG	94 °C, 30 s −55 °C, 30 s −72 °C, 30 s	313
		R: GCTTAATACAGTTACGATAG		

^(1)^ Amplification: denaturation-annealing-extension.

**Table 3:** Prevalence and contamination levels of *Campylobacter* in chicken and duck carcasses at wet markets in Korea during summer and winter.

Seasons	Sample	Prevalence (No. of Positive Samples/No. of Samples (%))	Contamination Level	
			No. of Positive Samples/No. of Samples (%)	Mean ± SD (CFU/g)
Summer	Chicken	7/80 (8.8)	3/80 (3.8)	32.1 ± 21.0
	Duck	15/80 (18.8)	7/80 (8.8)	15.7 ± 14.2
	Subtotal	22/160 (13.8)	10/160 (6.3)	20.6 ± 17.2
Winter	Chicken	8/72 (11.1)	19/72 (26.4)	20.4 ± 38.8
	Duck	15/74 (20.3)	38/74 (51.4)	427.4 ± 780.2
	Subtotal	23/146 (15.8)	57/146 (39.0)	301.1 ± 673.1
Total	Chicken	15/152 (9.9) ^A^	22/152 (14.5)	22.0 ± 36.6 ^b^
	Duck	30/154 (19.5) ^A^	45/154 (29.2)	366.1 ± 733.6 ^a^
	Total	45/306 (14.7)	67/306 (21.9)	259.8 ± 628.9

Different upper letters (A, a, and b) in the same column indicate a difference (*p* < 0.05).

**Table 4:** Percentage of susceptibility and resistance of seven antibiotics for *Campylobacter* isolates from poultry.

Class	Antibiotics	Susceptibility		Resistance	
		No. of Isolates	Ratio (%)	No. of Isolates	Ratio (%)
A ^(1)^	Amikacin	25	55.6	20	44.4
M	Erythromycin	43	95.6	2	4.4
T	Tetracycline	13	28.9	32	71.1
F	Ciprofloxacin	4	8.9	41	91.1
F	Enrofloxacin	38	84.4	7	15.6
Q	Nalidixic acid	3	6.7	42	93.3
Others	Chloramphenicol	45	100.0	0	0.0

^(1)^ A: Aminoglycosides, M: Macrolides; T: Tetracyclines; F: Fluoroquinolones; Q: Quinolones.

**Table 5:** Cytolethal Distending Toxin (CDT) gene profiles of *Campylobacter* isolated from chicken and duck carcasses at wet markets.

Toxin Profile	Number of Isolates				
	Chicken		Duck		Total (%)
	Summer	Winter	Summer	Winter	
Negative	1	-	2	1	4 (4.3)
*cdtA*+	-	-	-	-	-
*cdtB*+	-	-	-	-	-
*cdtC*+	-	-	-	-	-
*cdtA*+/*cdtB*+	-		1	-	1 (2.2)
*cdtA*+/*cdtC*+	-	-	1	-	1 (2.2)
*cdtB*+/*cdtC*+	1	4	1	1	7 (15.6)
*cdtA*+/*cdtB*+/*cdtC*+	5	4	10	13	32 (71.1)
Total	7	8	15	15	45 (100.0)
